# Optimisation of Scores Generated by an Online Feline Health–Related Quality of Life (HRQL) Instrument to Assist the Veterinary User Interpret Its Results

**DOI:** 10.3389/fvets.2020.601304

**Published:** 2021-01-06

**Authors:** Vinny Davies, Jacqueline Reid, E. Marian Scott

**Affiliations:** ^1^School of Computing Science, University of Glasgow, Glasgow, United Kingdom; ^2^School of Veterinary Medicine, University of Glasgow, Glasgow, United Kingdom; ^3^School of Mathematics & Statistics, University of Glasgow, Glasgow, United Kingdom

**Keywords:** health-related quality of life, cats, optimisation, health status, threshold, minimum important difference, interpretability, score normalization

## Abstract

Using methodology previously described for the dog health-related quality of life (HRQL) tool (VetMetrica™), the aim was to optimize the scores profile of a comparable feline online HRQL instrument for monitoring HRQL in cats, to assist in its interpretation. Measuring HRQL helps quantify the impact of disease and its treatment on well-being, aids clinical decision making and provides information in clinical trials. In Study 1, using data collected from previous studies, scores generated for three domains of HRQL (Vitality, Comfort, Emotional Well-being) in healthy cats were normalized using standard statistical techniques of logit transformation and *T*-scores, such that the average healthy cat has a score of 50 in all three HRQL domains. Using normalized scores from healthy and sick cats, a threshold score of 44.8 was determined, above which 70% of healthy cats should score. Study 2 determined the Minimal Important Difference (MID) in normalized score that constituted a clinically significant improvement in each domain. Three methods were tested in order to determine the MID, with the final choice made based on statistical and clinical considerations. Thresholds of 5, 7.5, and 5 were chosen for the three HRQL domains representing Vitality, Comfort and Emotional Well-being, respectively. This study makes available a means of displaying HRQL scores from an online application in an easily interpretable manner and quantifies a clinically meaningful improvement in score. To illustrate the practical application of these developments, three case examples are presented. Example 1 illustrates the raw and normalized scores for a group of overweight cats enrolled in a Feline Weight Management Programme. Example 2 shows three groups of osteoarthritic cats, each with different severity of disease. The third is an elderly, un-well cat whose HRQL was recorded over time, specifically to facilitate end of life discussion between owner and veterinary clinician.

## Introduction

In humans HRQL measurement is an important research area, quantifying the impact of disease and its treatment on an individual's daily well-being, to aid clinical decision making, provide an outcome measure in clinical trials and contribute to healthcare policy (1). The development of HRQL instruments for companion animals is growing and their value is increasingly recognized (2). HRQL instruments can be disease specific or generic (3–8). Generic instruments measure quality of life (QOL) in healthy or sick animals and are the only option when co-morbidities are present, as is the case with older animals. Profile rather than single item HRQL measures generate scores in multiple domains of HRQL, allowing for comprehensive analysis of HRQL changes over time, in contrast to a single item score which only tells us if an animal is better or worse.

Previously we reported the development, validation and reliability of web-based generic HRQL profile measures for the dog (9, 10) and the cat (11). They consist of simple behavioral questions (questionnaire items) for the owner (22 for the dog and 20 for the cat) that are scored on a 0-6 scale (0 = could not be less and 6=could not be more) and completed online in around 5 min. A list of these items which are either positive—for example “active,” or negative—for example “listless,” is available in Noble et al. (11). The dog tool generates scores in four domains of HRQL -Energetic/Enthusiastic (E/E), Happy/Content (H/C), Active/Comfortable (A/C), Calm/Relaxed (C/R)) compared with 3 for the cat–Vitality, Comfort, Emotional Well-being (EWB). These domains were derived using a multivariate statistical analysis called factor analysis (FA). Factor analysis is a technique that is used to reduce a large number of variables into fewer numbers of factors, in this case domains of HRQL. Details of this process for the dog and cat tools can be found in Reid et al.'s and Noble et al. (9–11), but briefly, using a test data set, several factor solutions are explored, each of which consists of a different number of factors. The optimum factor solution is the one that accounts for the most variability in the data, so for the dog, the four factor solution was optimum accounting for 72% of the variance (10) and in the cat the optimum solution contained three factors, accounting for 72.3% of the variance (11).

Although interpretability is a key element of a useful measurement scale, to date there is no agreement as to how HRQL scores should be presented to ensure the ease with which a user can interpret them. Users should be able to understand what an individual score produced by an instrument means, for example by comparing the score to a healthy population. They also need to know when any variation in scores is meaningful, for example is the treatment really working or is the disease really worsening as time goes on? This is currently an important research focus in the medical field (12), where according to these authors “the choice of what constitutes an important difference in a HRQL score will influence judgements about the success of a health care intervention, the required sample size of clinical studies, and the design of these studies.” Health—related quality of life instrument development is an expanding area in the veterinary arena and there is increasing appreciation of their value in measuring well-ness within a model of veterinary preventative healthcare and measuring the impact of chronic disease and its treatment (10). As a result, interpretability is equally as important in the veterinary as well as the medical field.

Currently, several techniques have been suggested to enhance interpretability of health measures for people including linking the scores to those of a specific population (norm-based scoring) such as a general population, populations with similar demographics or to a population with a specific disease (13). The scores profile of the dog tool was optimized to improve its interpretability by normalizing the scores to the age-related healthy dog population and deriving a threshold as a guide to the health of the dog (14). A detailed description of the rationale underlying the choice of these procedures can be found in Davies et al. (14). The significance of a change in score can be quantified through the calculation of a Minimum important difference (MID). This has been defined as “the smallest difference in score in the outcome of interest that informed patients or informed proxies perceive as important, either beneficial or harmful, and which would lead the patient or clinician to consider a change in the management” (15). The MID can be established using distributional and/or anchor-based techniques. A distribution-based approach relies on the statistical properties of the instrument and does not involve an external impression of change. Examples include effect size (16), normalized response mean (17) and the modified normalized response mean (18). On the other hand, anchor-based techniques use an external impression such as the patient's perception of a significant improvement or worsening of their condition to identify the change on the HRQL scale that corresponds to the MID (19). Such global measures of change are however strongly affected by the context in which they are used and subject to much variability making dependance on these problematic. Deyo and Centor (20) suggested that scales could be viewed as “diagnostic tests” for distinguishing improved patients from those that had not, with receiver operating characteristic (ROC) curves being utilized to describe a scale's ability to identify improvement. This more objective method to determining the MID using ROC was used in the dog study (14). Briefly, the characteristics of the test, namely sensitivity and specificity, which describe how well the test discriminates between two groups are calculated. Sensitivity describes how well a test identifies those with a particular disease (true positive) and specificity describes how well it correctly identifies those without that disease (true negative). The ROC curve plots sensitivity against 1—specificity and a cut-off (threshold value) is chosen above which cases are classified as positive while cases with scores below that cut-off are classified as negative. A test with perfect discrimination (no overlap in the diseased and non-diseased distributions: no false positives or false negatives) has a ROC curve that passes through the upper left corner of the ROC graph, providing 100% sensitivity and 100% specificity, but this is very rare. Therefore, the closer the ROC curve I to the upper left corner of the graph, the higher the overall accuracy of the test (21). In the dog study (14), the owner's impression of change (improved or unchanged) and the corresponding change in HRQL score were used to calculate a series of sensitivity and 1—specificity value pairs, which then made up the points on the ROC curve. Each point on the ROC curve was translated back to a value: a change in score. A point on the curve was chosen as the MID with due regard given to the consequences of the clinical implications of that choice.

The aim of this paper was to implement similar methods to those employed for the dog to improve the interpretability of the cat tool through normalization of scores, creation of a health status threshold and calculation of the MID.

## Study 1: Normalization and Creation of a Threshold

### Materials and Methods

#### Data

HRQL data for 107 healthy cats and 95 sick cats (Supplementary Material 3) were collected from University of Glasgow Small Animal Hospital (UGSAH), five general veterinary practices and one feline medicine specialist veterinary clinic, as part of previous HRQL instrument development studies, for which ethical approval was granted by the University of Glasgow Veterinary School. In the sick group there were no exclusion criteria and the only inclusion criterion was that the cats were suffering from a non-acute condition deemed likely to affect their QOL by the attending veterinary surgeon. The healthy cat group comprised cats deemed to be healthy by the attending veterinary surgeon.

#### Normalization Process Using Healthy Cats

Using raw owner-generated data from the 107 healthy cats (Supplementary Material 2), normalization was a two step process as follows.

**Step 1:** Transformation of raw 0−6 scores for each of the three HRQL domains to a continuous scale, using a logit transformation.

To allow the use of a logit transformation, HRQL scores (d) on the scale of 0−6 were converted to lie between 0 and 1, excluding exact 0 and 1 values. This was achieved by adding an arbitrary value, 0.1, at each end of the scale and dividing by 6.2 (the maximum score after the arbitrary values has been plus 0.1) as follows: $d^{\prime }=\frac{d+\;0.1}{6.2}.$

Thereafter these converted scores were *logit* transformed to the continuous real scale (values between very large negative and very large positive values) as follows: $d^{\prime \prime }=\;logit\left(d^{\prime }\right)=\log\left(\frac{d^{\prime }}{1-d^{\prime }}\right)$. The transformation to a *logit* scale puts the measurement on a continuous scale, which is standard statistical practice in the testing literature (22).

**Step 2:** Following transformation to a continuous distribution, *T*-scores were calculated for 3 HRQL domain scores based on the sample means (μ), and sample SDs (σ), of the scores as follows: $T=\frac{d^{\prime \prime }-\;\mu }{\sigma \;}$.

The *T*-scores have mean 0 and standard deviation 1. Finally, *T*-scores were scaled by multiplying them by 10 and adding 50, thus providing easily interpretable scores, where a score of 50 represented the healthy population norm for a given HRQL domain *S* = 10 × *T* + 50.

In combination, these steps produce scores that are comparable across all HRQL domains, with the distributions for each set of scores having the same mean (50) and standard deviation (10).

#### Creation of a Threshold to Distinguish Healthy From Sick Cats

To allow the choice of a threshold which was consistent across all HRQL domains, with each domain having the same mean and standard deviation, a density plot of the theoretical distribution of the healthy cat population was constructed and the HRQL score representing the 30th percentile identified. Thereafter boxplots of the normalized HRQL domain scores (Vitality, Comfort and Emotional Well-being) for sick and healthy cats were constructed and examined visually to establish if the chosen threshold was effective at separating sick and healthy populations.

#### Examples Used to Demonstrate the Practical Application of Score Normalization and Threshold Creation

Examples were drawn from 3 previous studies carried out using the HRQL scale.

Example 1 illustrates the raw and normalized scores for a group of overweight cats enrolled in a Feline Weight Management Programme. Example 2 shows three groups of osteoarthritic cats, each with different severity of disease. The third example is the HRQL profile of an elderly, un-well cat whose HRQL was recorded over time, specifically to facilitate end of life discussion between owner and veterinary clinician.

### Results

#### Data

The mean age +/– standard deviation of the cats was 6.9 +/– 2.96 (range <1–11 years) and 11.1 +/– 4.25 (range <1–20 years) for healthy and sick cats, respectively. There was no significant difference between the groups (Two–Sample *T*–Test, *p* = <0.001). There were 55 males and 52 females in the healthy group and 47 males and 48 females in the sick group. The majority of cats were domestic shorthair.

#### Normalization Process Using Healthy Cats

**Figure 1:**
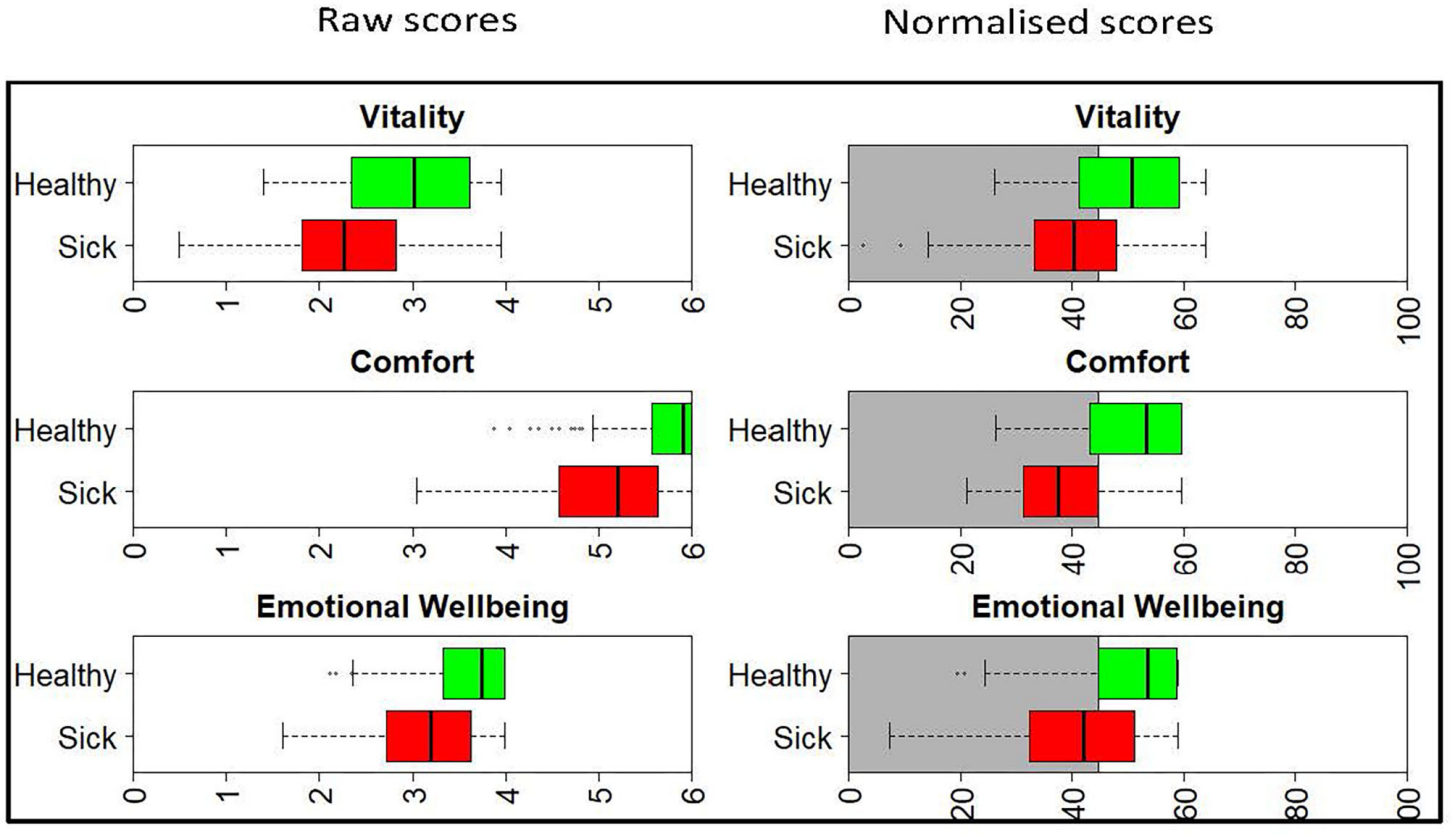
Boxplots showing the raw and normalized HRQL domain scores for sick (red) and healthy (green) cats. The threshold for the normalized scores is set at 44.8 for all domains, with 70% of healthy cats estimated to score above this threshold. The shaded gray area represents the area below the 44.8 threshold score.

The left panel of Figure 1 shows the raw score distribution for the sick (red) and healthy (green) cats. There is considerable overlap in the Vitality and EWB domains, but less so in the Comfort domain. In the Comfort domain the boxplot for the healthy cats is non-symmetrical, with no tail on the right-hand side. This is clear evidence of a ceiling effect in the healthy cat group, with a high percentage of owners (42 of 107) recording a maximum score of 6. The right panel shows the scores once normalized. Now, because all scores are presented relative to the average healthy dog (score of 50), the domains are directly comparable to each other.

#### Establishing Thresholds Between Healthy and Sick Cats

**Figure 2:**
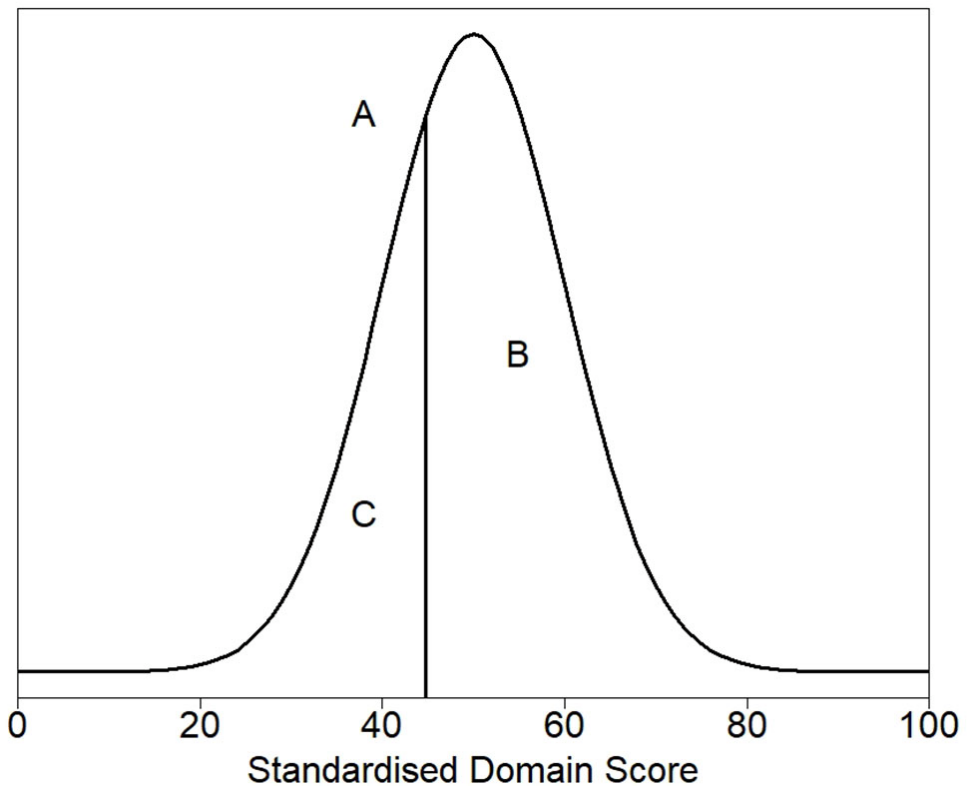
Density plot of the theoretical distribution of the healthy cat population. **(A)** denotes the threshold for dividing healthy and sick cats, the 30th percentile (a HRQL score of 44.8). Above this threshold (to the right of the plot) are 70% of the healthy cat population predicted to be healthy, the area marked by **(B)**. Below the threshold (to the left of the plot) are 30% of the healthy population predicted to be sick, the area marked by **(C)**.

Figure 2 shows the density plot of the theoretical distribution of the healthy cat population. Marked on the plot is (A), a vertical line denoting the threshold for dividing healthy and sick cats, the 30th percentile (a HRQL score of 44.8). Above this threshold (to the right of the plot) are 70% of the healthy cat population predicted to be healthy, the area marked by B. Below the threshold (to the left of the plot) are 30% of the healthy population predicted to be sick, the area marked by C. The right panel of Figure 1 shows the normalized scores for healthy and sick cats with the shaded gray area representing the area below the 44.8 threshold score, and although the normalized scores for the sick and healthy cats overlap, this was considered acceptable at the 44.8 cut-off point.

#### Practical Applications

**Figure 3:**
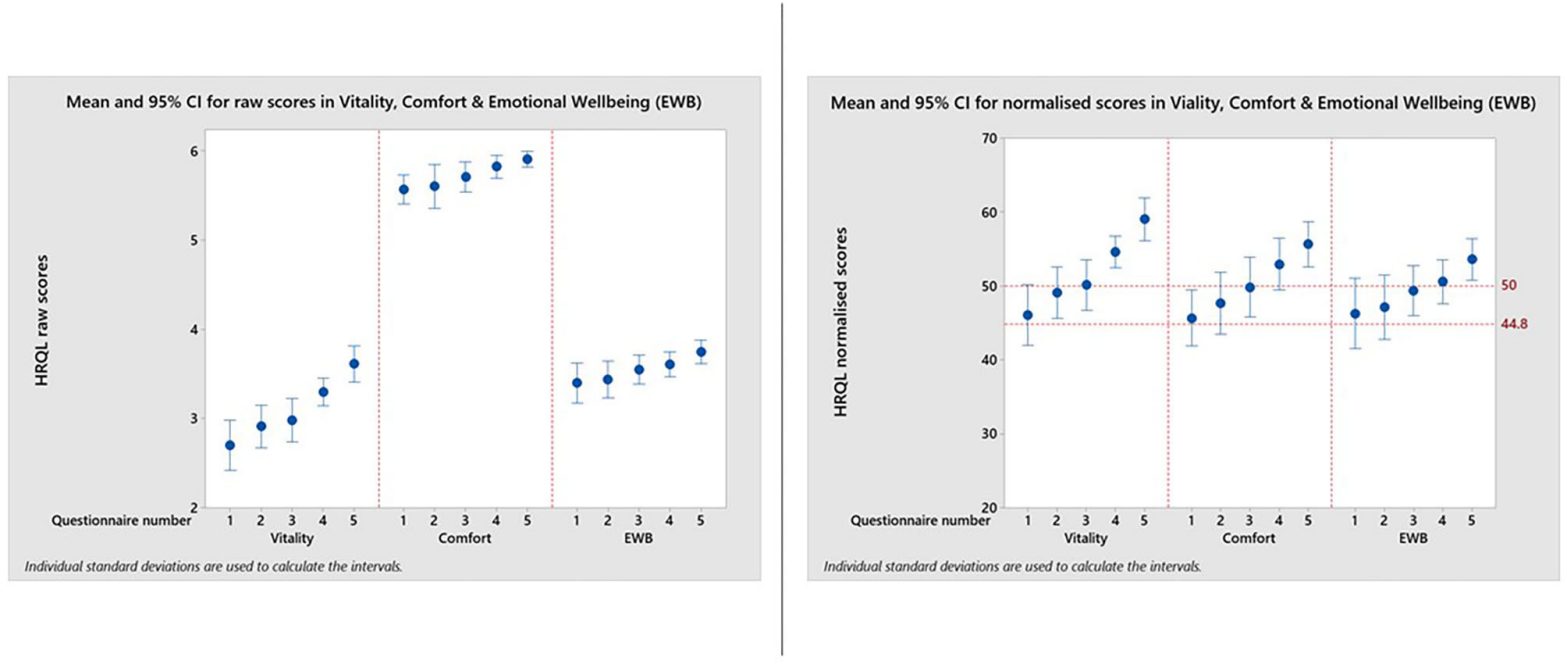
Reproduced with kind permission of Dr. Barr Hadar, Ontario Veterinary College, University of Guelph. Means and 95% confidence intervals for HRQL scores in Vitality, Comfort, Emotional Well-being for a group of 21 overweight cats enrolled on a weight loss programme whose owners completed a baseline score before treatment and then four assessments at approximately monthly intervals. A, Raw domain scores; B, Normalized domain scores. A score of 50 = the average healthy cat score and 70% of healthy cats will score above the 44.8 threshold.

Figure 3 and Table 1 show how the appearance of the raw and normalized scores differed in a group of 21 overweight cats enrolled in a Feline Weight Management Programme, where owners completed one assessment before treatment to provide baseline data, then four assessments at approximately monthly intervals following treatment. Formal analysis using a one-way ANOVA showed, for raw scores, *p* = <0.001 for Vitality, *p* = 0.021 for Comfort and *p* = 0.043 for EWB and for normalized scores *p* = <0.001 for Vitality, *p* = 0.001 for Comfort and *p* = 0.045 for EWB, confirming a trend for improvement over time for all domains. In the raw scores the improvement was clearest in the Vitality domain, but with the normalized scores the improvement was equally clear in all three domains. Again there is evidence of a ceiling effect in the Comfort domain where the maximum score in questionnaires 1–5 was 6 (Table 1). However, despite this ceiling effect, the normalized scores for each domain are much more readily interpretable, both in relation to each other and to the chosen common threshold value of 44.8.

**Table 1:** Descriptive statistics for all three HRQL domain scores both raw (A) and normalized (B), for questionnaires 1−5 completed by owners of a group of 21 overweight cats enrolled on a weight loss programme.

**Domain**	**Questionnaire No**	**Mean**	**SD**	**Minimum**	**Q1**	**Median**	**Q3**	**Maximum**
**(A)**								
Vitality—Raw score	1	2.70	0.62	1.33	2.26	2.74	3.19	3.82
	2	2.91	0.53	1.50	2.60	3.05	3.30	3.54
	3	2.98	0.54	1.87	2.62	3.02	3.35	3.95
	4	3.30	0.34	2.46	3.09	3.33	3.54	3.78
	5	3.61	0.43	2.42	3.32	3.81	3.89	3.95
Comfort–Raw score	1	5.57	0.35	4.58	5.34	5.57	5.87	6.00
	2	5.61	0.54	3.97	5.54	5.78	5.94	6.00
	3	5.71	0.38	4.82	5.59	5.84	6.00	6.00
	4	5.83	0.28	4.87	5.78	5.94	6.00	6.00
	5	5.91	0.19	5.34	5.91	6.00	6.00	6.00
EWB—Raw score	1	3.40	0.50	2.47	3.05	3.38	3.99	3.99
	2	3.44	0.46	2.30	3.18	3.39	3.87	3.99
	3	3.55	0.35	2.74	3.33	3.54	3.86	3.99
	4	3.61	0.31	3.02	3.37	3.69	3.78	3.99
	5	3.75	0.27	3.06	3.59	3.75	3.99	3.99
**(B)**								
Vitality—Normalized	1	46.08	8.94	24.85	40.09	46.83	52.97	62.01
	2	49.10	7.64	28.00	44.96	51.14	54.57	57.98
	3	50.15	7.58	34.29	45.18	50.79	55.25	63.99
	4	54.60	4.73	43.03	51.62	54.96	57.81	61.41
	5	59.06	6.01	42.39	54.82	61.89	63.09	63.99
Comfort–Normalized	1	45.65	8.29	31.49	39.38	43.24	51.51	59.55
	2	47.67	9.23	27.00	42.61	48.28	55.04	59.55
	3	49.86	8.85	33.62	41.48	50.47	59.55	59.55
	4	52.97	7.75	34.01	48.25	54.82	59.55	59.55
	5	55.69	6.38	39.37	53.45	59.55	59.55	59.55
EWB—Normalized	1	46.28	10.45	27.14	38.98	45.73	58.84	58.84
	2	47.11	9.57	23.40	41.62	46.06	56.26	58.84
	3	49.35	7.43	32.74	44.72	49.19	55.89	58.84
	4	50.60	6.55	38.32	45.56	52.38	54.31	58.84
	5	53.62	5.79	39.31	50.13	53.71	58.84	58.84

**Figure 4:**
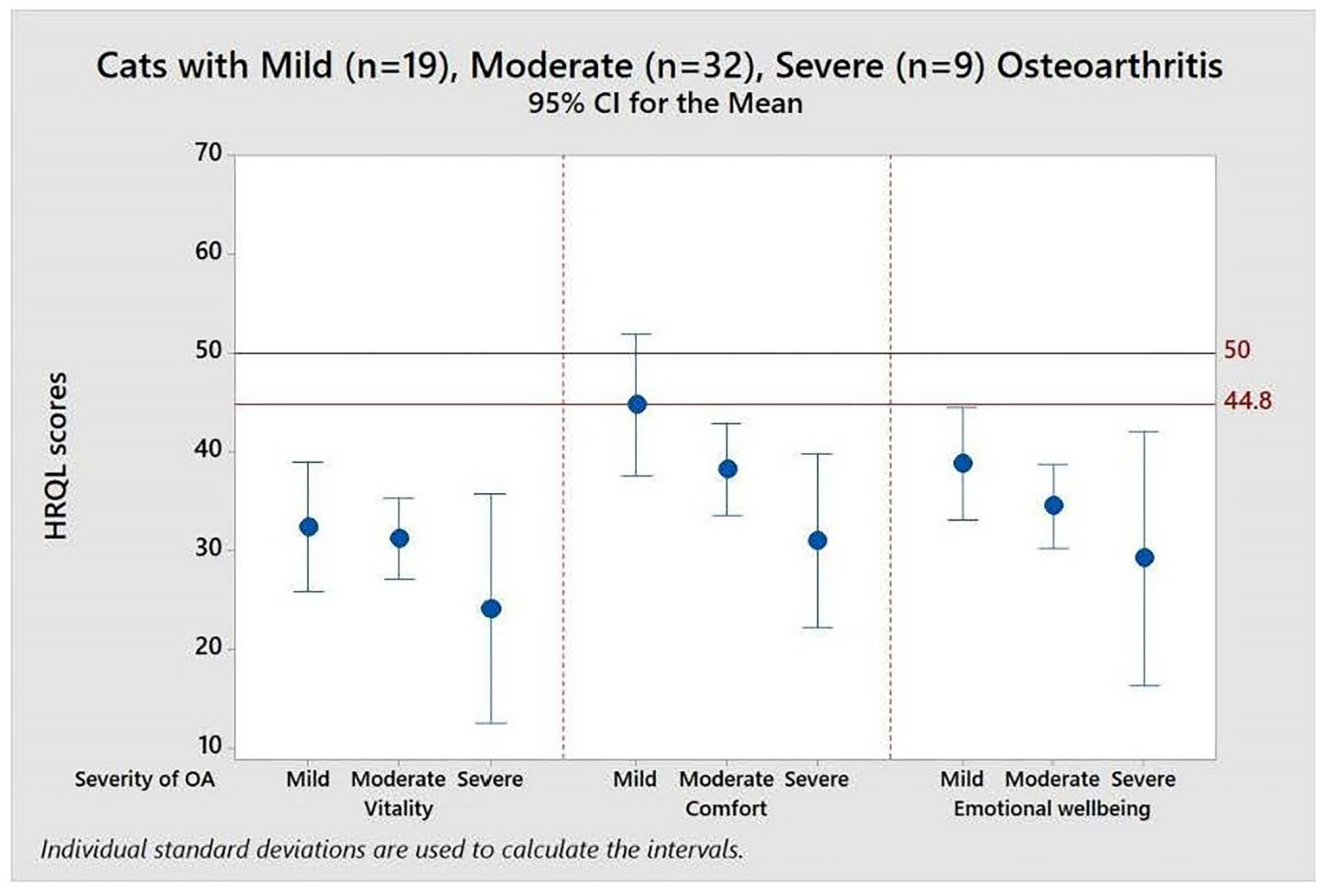
Means and 95% confidence intervals for HRQL scores in Vitality, Comfort, Emotional Well-being for a group of 60 cats with varying severity (mild, moderate, severe) osteoarthritis. A score of 50 = the average healthy cat score and 70% of healthy cats will score above the 44.8 threshold.

Figure 4 demonstrates the value of having the reference scores (50 and 44.8) when interpreting the impact of different levels of disease severity, in this case in a cohort of cats with osteoarthritis (OA), classified as mild, moderate or severe by the attending clinician. The mean scores for all groups of cats were below that of the average healthy cat for all domains indicating that OA impacted the quality of life of cats even when only mildly affected by the disease.

Table 2 shows the descriptive statistics for all three HRQL domains for mild, moderate and severely affected OA cats. Formal analysis using a one-way ANOVA showed *p* = 0.631 for Vitality, *p* = 0.010 for Comfort and *p* = 0.325 for EWB. Although only Comfort was statistically significant, the mean HRQL scores show a trend which support that the HRQL scores decline with OA severity (mild > moderate > severe).

**Table 2:** Descriptive statistics for all three HRQL domains for mild, moderate and severely affected OA cats.

**Domain**	**Severity**	**Mean**	**SD**	**Minimum**	**Q1**	**Median**	**Q3**	**Maximum**
Vitality	Mild (*n* = 19)	32.35	13.68	11.86	20.89	30.14	43.01	56.10
	Moderate (*n* = 32)	31.19	11.34	9.20	24.01	31.60	39.91	52.03
	Severe (*n* = 9)	24.06	15.06	0.05	15.31	20.31	37.00	49.38
Comfort	Mild (*n* = 19)	44.75	14.90	22.02	31.90	44.80	57.01	70
	Moderate (*n* = 32)	38.12	12.92	23.26	30.38	34.10	41.61	70
	Severe (*n* = 9)	30.89	11.43	15.71	22.76	26.10	43.61	47.56
EWB	Mild (*n* = 19)	38.72	11.83	14.00	29.50	38.80	48.71	58.80
	Moderate (*n* = 32)	34.43	11.81	0.31	30.05	35.90	43.56	52.32
	Severe (*n* = 9)	29.16	16.78	0.00	17.94	29.14	40.39	58.80

**Figure 5:**
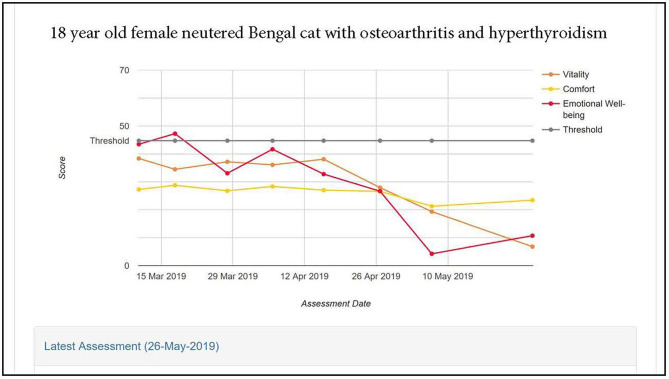
HRQL scores in Vitality, Comfort and Emotional Well-being for an 18 year old female neutered Bengal cat with osteoarthritis and hyperthyroidism. A score of 50 = the average healthy cat score and 70% of healthy cats will score above the 44.8 threshold.

Figure 5 illustrates the scores profile for an 18-year-old Bengal female neutered cat with OA, hyperthyroid and controlled hypertension, recorded over a 2-month period. At enrolment all three domain scores were below the healthy cat average. There was a steady decline in Emotional Well-being scores over the period, but scores for Comfort and Vitality domains were stable until week 5, after which they declined, Vitality showing more deterioration than Comfort.

## Study 2: Calculating the Mid for an Improvement in the Normalized HRQL Scores

### Materials and Methods

#### Data

Data were obtained from previous studies as before for a mixed (healthy and unhealthy) group of 95 cats (Supplementary Material 4) with HRQL scores collected on two or more occasions from Glasgow University Small Animal Hospital, Liverpool University Small animal Practice, five veterinary practices in the UK, one in Canada and Australia and three in the US. In addition to the 20 questions comprising the feline VetMetrica assessment, owners were asked to record whether they believed their cat's health had improved, stayed unchanged or worsened since the previous assessment. Of the 95 cats, data from two cats was incomplete, owners of 29 cats considered health status had improved, 58 had not seen a change and six considered their cat's health had deteriorated. These six cats were removed based on their small number. The unchanged group of 58 cats contained cats that were both healthy and unhealthy.

#### Calculation of Possible MIDs

Only the 1st and 2nd second assessments from each cat were used. For each cat, the difference between the normalized scores for assessments 1 and 2 for each domain was calculated, and generally these followed a normal distribution. The mid-point between the difference in normalized scores for each domain was selected and using the corresponding owner impression of change (unchanged or improved health) the sensitivity and specificity for each possible MID (mid-point) was calculated. For each HRQL domain, the sensitivities were then plotted against the corresponding 1—specificities to create the ROC curves for each HRQL domain (14).

#### Selection of MIDs

The methodology is reported by Davies et al. (14) and can be found reproduced with permission in the Supplementary Material 1. Briefly, using ROC curves and all possible calculated MIDs, several different methods to calculate the most appropriate MID for each HRQL domain were considered. A ROCconsistent method as described by Davies et al. (14), where all domains have the same MID followed by a similar method which allowed for different MIDs in different domains (ROCdomain) was used. Finally a set of MIDs (VetMetrica Cat) were chosen based on their position on the ROC curve.

### Results

**Figure 6:**
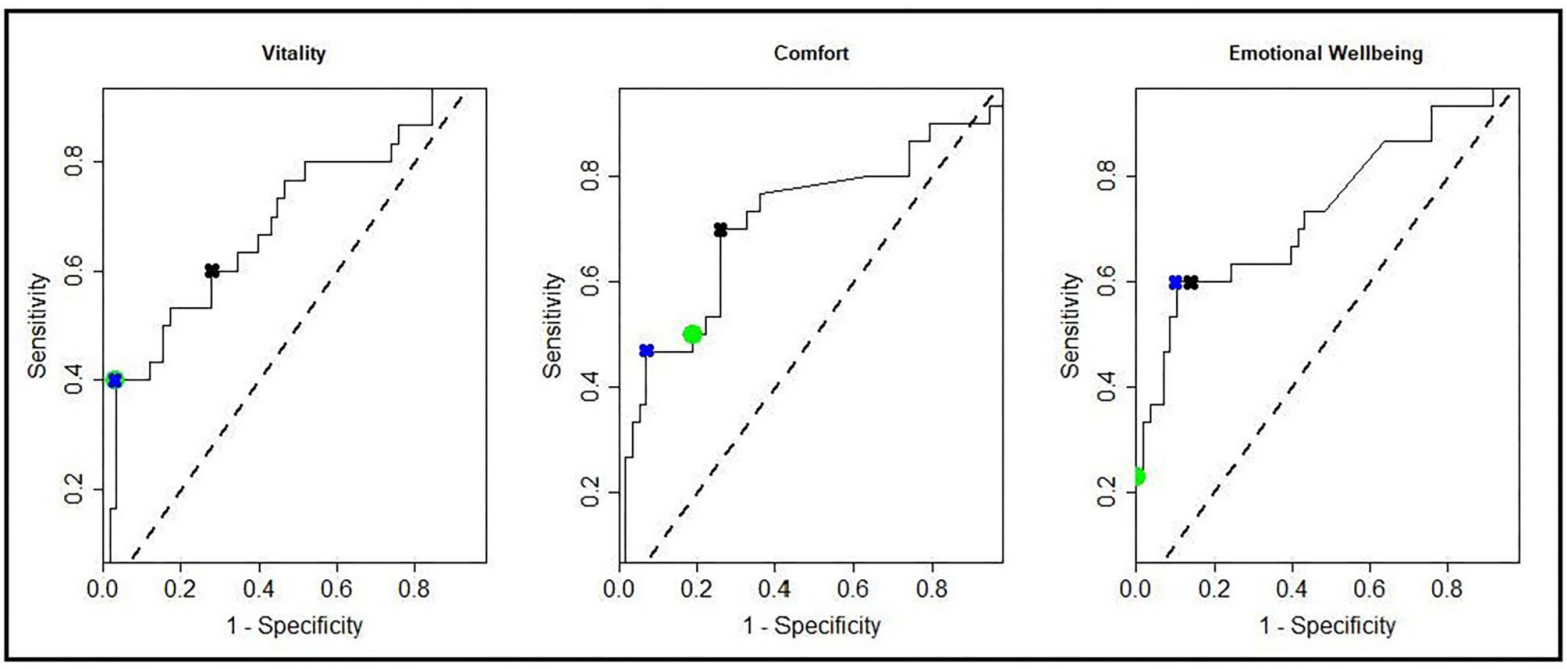
ROC curves showing all possible MIDS for each of the HRQL domains; Vitality, Comfort, Emotional Well-being. Marked on the ROC curves are the sensitivities and 1—specificities for the MIDconsistent method (green circles), MIDdomain method (blue crosses), and the MIDs used in the cat section of the VetMetrica application (black crosses).

**Figure 7:**
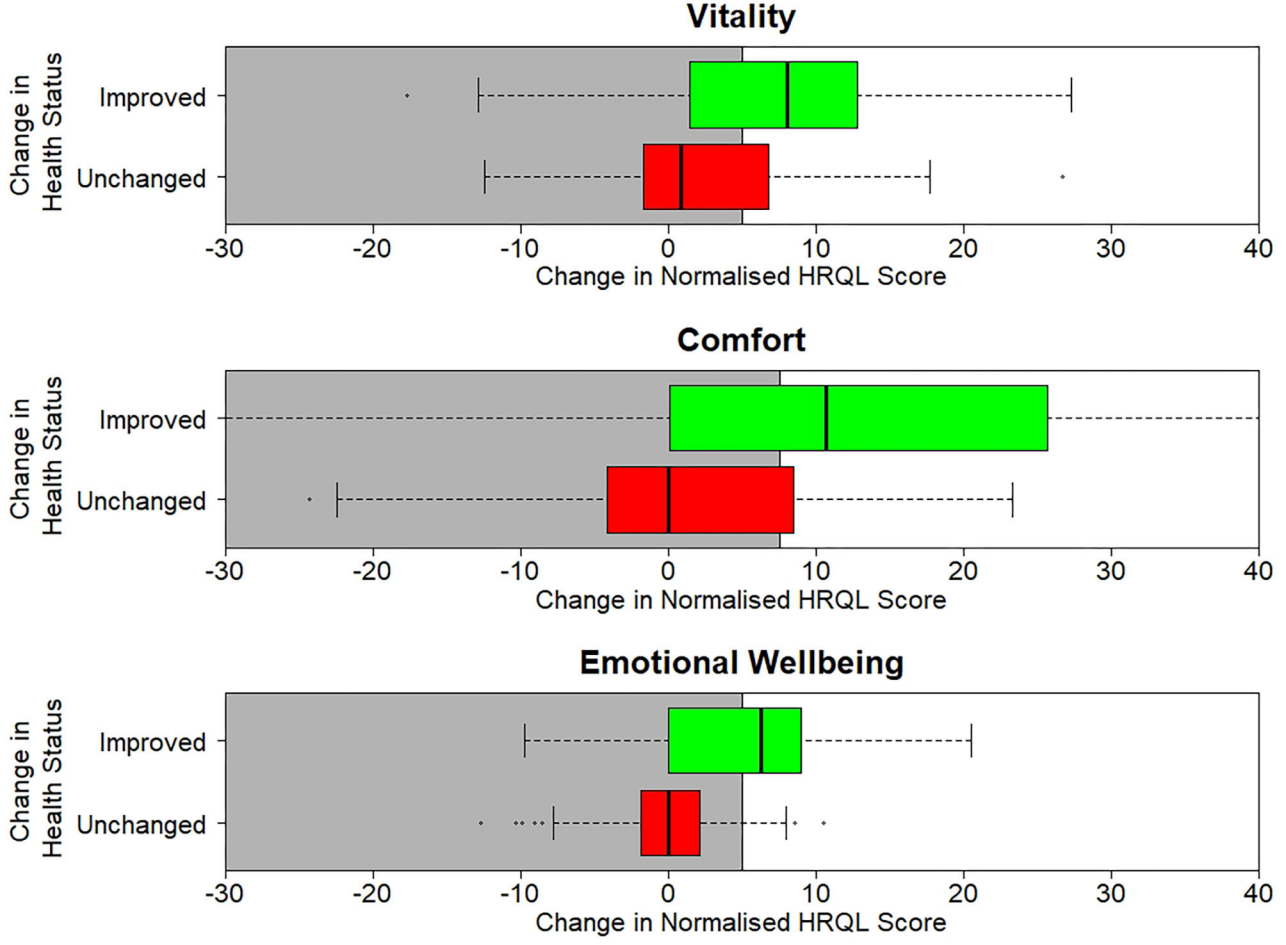
Boxplots of the change in normalized scores in each of the three normalized HRQL domains, Vitality, Comfort and Emotional Well-being. For each domain there are two boxplots, one for cats whose health is unchanged (red) and one where their health has improved (green).

Table 3 shows the sensitivity, specificity and classification accuracy for ROCconsistent, ROCdomain and VetMetrica Cat methods. Figure 6 shows the ROC curves constructed for each HRQL domain as well as the corresponding sensitivities and 1—specificities. The ROC curves show the sensitivity and specificity trade off that must be considered when choosing the final MIDs. After considering the clinical implications from the different options displayed in Table 3 and Figure 6, the chosen MID values for VetMetrica Cat were 5, 7.5, and 5, respectively for the Vitality, Comfort and EWB HRQL domains. Figure 7 shows boxplots of the change in normalized HRQL domains scores. For each domain, separate boxplots are given for cats whose owners reported an improvement in health (green) and no change in health (red). On each boxplot the area below the MIDs are shaded, with the MIDs being 5, 7.5, and 5, respectively for the Vitality, Comfort and EWB HRQL domains. In each case the boxplots show an acceptable demarcation between the cats who have improved in health and those that have remained unchanged.

**Table 3:** The sensitivities, specificities and classification accuracies of MIDconsistent, MIDdomain and VetMetrica Cat methods of MID calculation for HRQL domains Vitality, Comfort and Emotional well-being (EWB).

**Domain**	**Method**	**MID**	**Sensitivity**	**Specificity**	**Accuracy**
Vitality	MIDconsistent	10.8	0.40	0.97	0.77
Vitality	MIDdomain	10.8	0.40	0.97	0.77
Vitality	VetMetrica Cat	5	0.60	0.72	0.68
Comfort	MIDconsistent	10.8	0.50	0.81	0.70
Comfort	MIDdomain	15.5	0.47	0.93	0.77
Comfort	VetMetrica Cat	7.5	0.70	0.74	0.73
EWB	MIDconsistent	10.8	0.23	1	0.74
EWB	MIDdomain	5.4	0.60	0.90	0.80
EWB	VetMetrica Cat	5	0.60	0.86	0.77

## Discussion

Previously we have described three strategies to enhance the interpretability of a web-based, generic, profile HRQL measure for the dog (VetMetrica) (14) and this paper describes how these, namely score normalization, health status threshold and MID, were applied to the feline VetMetrica instrument. Norm-based scoring algorithms transformed the raw scores such that, on a 0–100 continuous scale, 50 represented the average healthy cat with a standard deviation of 10, thus scores above 50 are better than average and those below are worse compared to the healthy population. In the dog HRQL scores were normalized to the average healthy dog, according to two age groups, 0–≤7 yrs and ≥8 yrs. However, for the cat there were insufficient data to form similar age groups. Whereas, the authors were concerned that it was unrealistic to have a 1 year old dog in the same group as one that was 7 years old (14), subsequent work investigating the impact of age, breed and sex on QOL has shown that the decline in QOL with age in healthy dogs is very slow (in press). For example the decline in the score for Energetic/Enthusiastic over a 12 month period was 0.05. Clearly it would be inappropriate to extrapolate these findings to the cat, but in general it may not be as important to divide subjects into different age groups as was once thought.

The normalization process does not take account of the ceiling effects which occur when scores reach the maximum as a result of high numbers of healthy subjects scoring very highly. In the dog ceiling effects were seen in “Active/Comfortable” and “Happy/Content” domains which reflect physical and emotional well-being, respectively, but in the cat only occurred in the “Comfort” domain which reflects physical well-being. Many people accept that cats may appear less expressive than dogs when it comes to their emotions, and therefore the behaviors making up the EWB domain may be less overt than their equivalent in the dog. Accordingly, this may have been a contributing factor to the lack of ceiling effect in the EWB domain.

Several methods, both parametric and non-parametric, have been proposed to deal with ceiling effects (23, 24), but due to the large number of cats that achieved a maximum score in the “Comfort” domain it was considered that these would not be effective at correcting the skewness of these data. Accordingly, it was decided to follow the standard statistical practice of transforming data to a continuous scale (22), and then calculating norm-based scores that are comparable across all the domains (25). It is important to note that the difference between normalized scores generates approximately normally distributed data, so ceiling effects do not affect the calculation of a MID.

A comparison of the raw score profile for a group of overweight cats recruited to a weight loss programme with the normalized profile (Figure 3) demonstrates the superior interpretability of normalized scores. Whereas, there is no reference point for the raw scores, the score of 50 for the average healthy cat and the threshold of 44.8 above which 70% healthy cats will score provide a useful reference point which also allows a direct comparison between domains, which are now presented on the same metric. At baseline the HRQL of the overweight cats is below average in all three domains, although scores are above the 70% threshold. By questionnaire 3 (2 months into the weight loss programme) they have improved such that the mean of the group is equivalent to the average healthy cat. That improvement is then sustained until the end of the trial. Furthermore, the two case studies presented (Figures 4, 5) demonstrate how the normalized scores and threshold provide the veterinary surgeon with an immediate visual interpretation of individual or group scores relative to health status over time. Notably, many owners of cats with OA don't recognize the signs of mild disease, and yet, at that level, the condition has a marked impact on all domains of QOL, especially Vitality and EWB (Figure 4). In the individual cat (Figure 5), the physical and emotional impacts of disease follow a different trajectory, with EWB (emotional impact) declining steadily from the start of screening compared with Vitality and Comfort (physical impact) which remain stable before declining sharply at different time points. This ability to distinguish emotional from physical impact is one of the advantages of a profile measure compared with a single item score which only tells us whether a patient is better or worse, but no more. On a day to day basis there will be natural variation in domain scores and so it is important to distinguish clinically significant change from “noise.” It is important to be able to advise owners of healthy cats that some change is within normal parameters, and unlikely to indicate any health change. Conversely, the clinician needs to know when an improvement in scores represents a positive effect of treatment in the sick cat. This is the function of the MID. It is equally important to determine if a deterioration in scores is meaningful, but unfortunately there were insufficient data available to investigate this. Several factors may have contributed to this. Owners may have habituated to their cat's condition and believed them to be unchanged. Alternatively they may not have remembered their cat's previous health status accurately (recall bias). In any study, this bias can be more significant when the participant has a poor memory in general and when the interval between events is long. Other factors that can influence it include age, education, socioeconomic status and the importance of the outcome to the respondent. Regarding the latter, owners may be reluctant to admit their cat has deteriorated (social desirability bias) and so it may be that some “unchanged” cats were in fact worse. The authors accept that this is a significant limitation to the use of the scale which will be addressed as more data become available. The sick cats used in this study were suffering from a variety of chronic diseases expected to impact their QOL which was appropriate for a generic scale. However, it is important to appreciate that, like validity, the MID is not an inherent property of the scale, but a feature of the scale as it is used in a particular clinical context. Accordingly, the MIDs of 5, 7.5, and 5 calculated for Vitality, Comfort and EWB, respectively, in this study for a general population may not apply when the scale is used in disease specific populations.

Small sample sizes limited the scope of these studies. While the available data were considered adequate for the normalization to the healthy cat population, it may be that experience with the tool will demonstrate that age should also be incorporated in the normalization process, as it was in the dog. Furthermore, the lack of a MID for deterioration as well as improvement is a limitation to its use. However, it is not uncommon for existing tools to undergo a continual process of refinement to accommodate new populations and contexts in which they are to be used (26).

In conclusion, if a measurement instrument is not easily interpreted, it is of limited use in clinical practice and research. This work substantially improves the interpretability of the VetMetrica generic HRQL instrument for the cat and will contribute to the body of knowledge regarding the impact of chronic disease on the emotional and physical health of this enigmatic species.

## Data Availability Statement

The original contributions generated for this study are included in the article/Supplementary Materials, further enquiries can be directed to the corresponding author.

## Ethics Statement

The original animal study was approved by the University of Glasgow Ethics and Welfare Committee and this retrospective data analysis by the RCVS Ethics Review Panel. Written informed consent was obtained from the owners for the participation of their animals in this study.

## Author Contributions

VD, JR, and MS: conceptualization, writing, review, and editing. JR: data curation and original manuscript. VD and MS: statistical analysis. All authors contributed to the article and approved the submitted version.

## Conflict of Interest

JR was employed by the company NewMetrica Ltd. The remaining authors declare that the research was conducted in the absence of any commercial or financial relationships that could be construed as a potential conflict of interest.

## Supplementary Material

Supplementary Material 1 Calculating the MID for improvements in the normalised scores.

Supplementary Material 2 Profile of scores for 107 healthy cats.

Supplementary Material 3 Profile of scores for 202 sick and healthy cats.

Supplementary Material 4 Profile of scores for the 1st and 2nd assessments from 95 sick and healthy cats.
