# Supplementary material for: Optimisation of Scores Generated by an Online Feline Health–Related Quality of Life (HRQL) Instrument to Assist the Veterinary User Interpret Its Results
Source: Front Vet Sci. 2021 Jan 6;7:601304. doi: 10.3389/fvets.2020.601304 (PMC7815521; doi:10.3389/fvets.2020.601304)
Supplement: Supplementary Material 4 — Profile of scores for the 1st and 2nd assessments from 95 sick and healthy cats. [file Data_Sheet_1.docx]

**Calculating the MID for an improvement in the normalised HRQL scores**

The choice of methodology to calculate the MID centred on a multi-step strategy, based on the authors’ experience with the dog tool in which two methods were investigated, namely ROCdomain (separate MID for each domain) and ROCconsistent (single MID for all domains), with the latter considered optimal^14^. For the cat, the ROCconsistent method provided a single MID which could be used across each HRQL domain, but was considerably greater than that of the dog (10.8 compared with 7.7). A MID of 10.8, provided high specificity, but relatively low sensitivity, more so in EWB compared with Vitality and Comfort. The MID could be reduced to increase sensitivity, but would require a decrease of different amounts in each domain to balance performance in terms of sensitivity and specificity. The ROCdomain method resulted in MIDs of 10.8, 15.5 and 5.4 for Vitality, Comfort and EWB respectively which, considering the disparity in the values, suggested that each domain would require a different MID. However, visual inspection of the ROC curve for the Comfort domain in Figure 5 shows another point on the ROC curve which balances sensitivity and specificity reasonably well and is closer to the top left corner of the graph. Similarly, the ROC curve for Vitality shows that there are other MIDs which are approximately the same distance from the top left corner, but which more evenly balance sensitivity and specificity. Because of the inconsistent findings for ROCconsistent and ROCdomain methods when applied to the feline tool, the final MIDs chosen for VetMetrica Cat were 5, 7.5 and 5 respectively for the Vitality, Comfort and EWB domains. This was based on the ROCdomain method with the MIDs in Vitality and Comfort reduced in order to better balance sensitivity and specificity. For EWB, the MID derived using the ROCdomain method (5.4) was rounded to 5 which decreased performance slightly, but increased usability. For the Vitality domain, there were a number of MIDs around 5 that performed similarly in terms of sensitivity and specificity so an MID of 5 was chosen to be consistent with EWB. The point that was closest to the top left corner of the ROC curve was chosen as the MID for Comfort. Generally the values chosen resulted in an acceptable balance of sensitivity and specificity, while maintaining some consistency between domains to enhance the user experience. Although the slightly lower sensitivity than specificity provided by VetMetrica Cat MID means that a number of cats will be diagnosed as having not changed when in fact they have improved, on balance this makes clinical sense. It errs on the side of caution in terms of not diagnosing cats as improved when they have not. Overall, the results of Table 1 (main manuscript) represent useful levels of accuracy providing that those using the instrument and interpreting its output understand the limits of its accuracy and use their clinical judgement accordingly.
